# Supplementary material for: Local colonisations and extinctions of European birds are poorly explained by changes in climate suitability
Source: Nat Commun. 2023 Jul 20;14:4304. doi: 10.1038/s41467-023-39093-1 (PMC10359363; doi:10.1038/s41467-023-39093-1)
Supplement: Supplementary file 1 — Supplementary Information [file 41467_2023_39093_MOESM1_ESM.pdf]

Local colonisations and extinctions of European birds are poorly explained by changes in climate suitability

Supplementary Information, Figures, and Tables

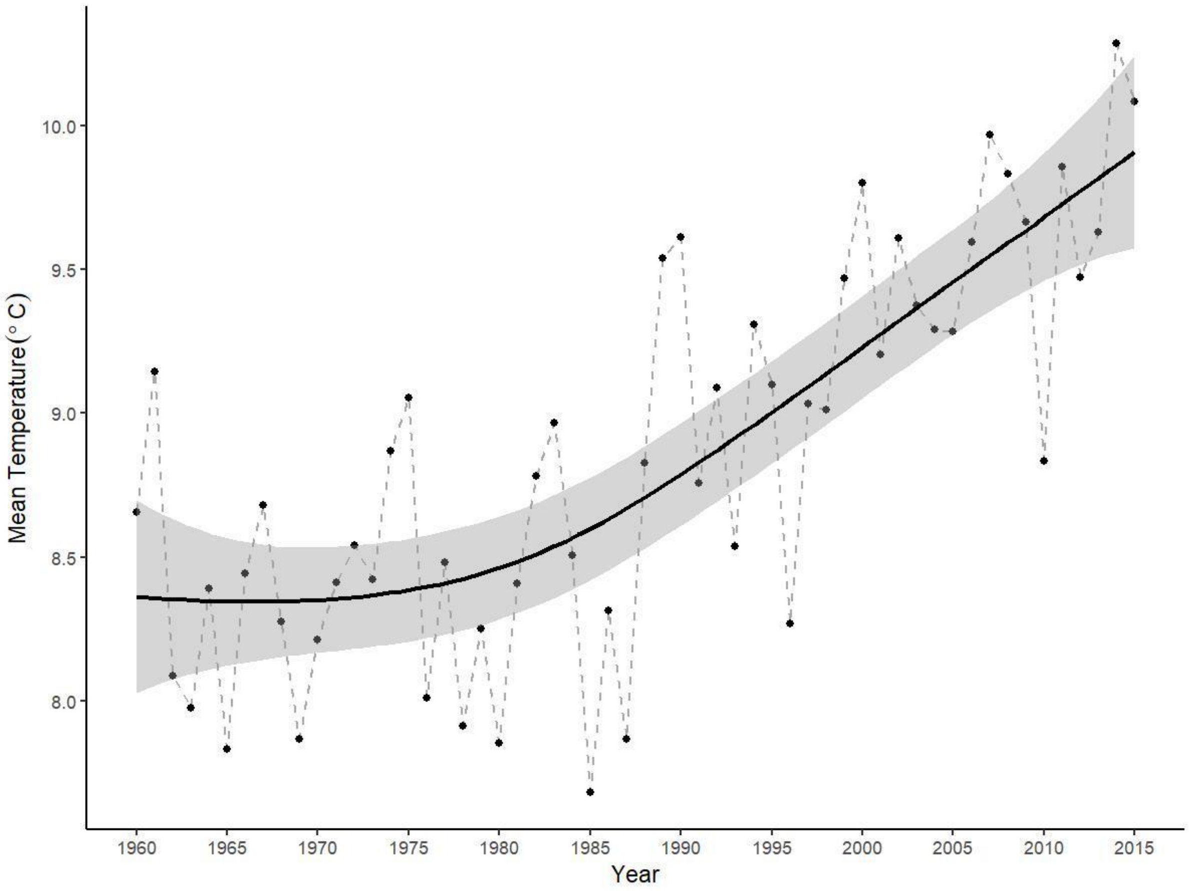

Supplementary Figure S1: Mean Temperature over the study area between 1960 and 2015. The points and dashed line indicate the mean annual observed temperature across all of Europe calculated using the CRS TS 3.25 0.5° dataset. The black line indicates the smoothed predictions from a GAM, with the shaded areas indicating the 95% confidence intervals around this model. Source data are provided as a Source Data file.

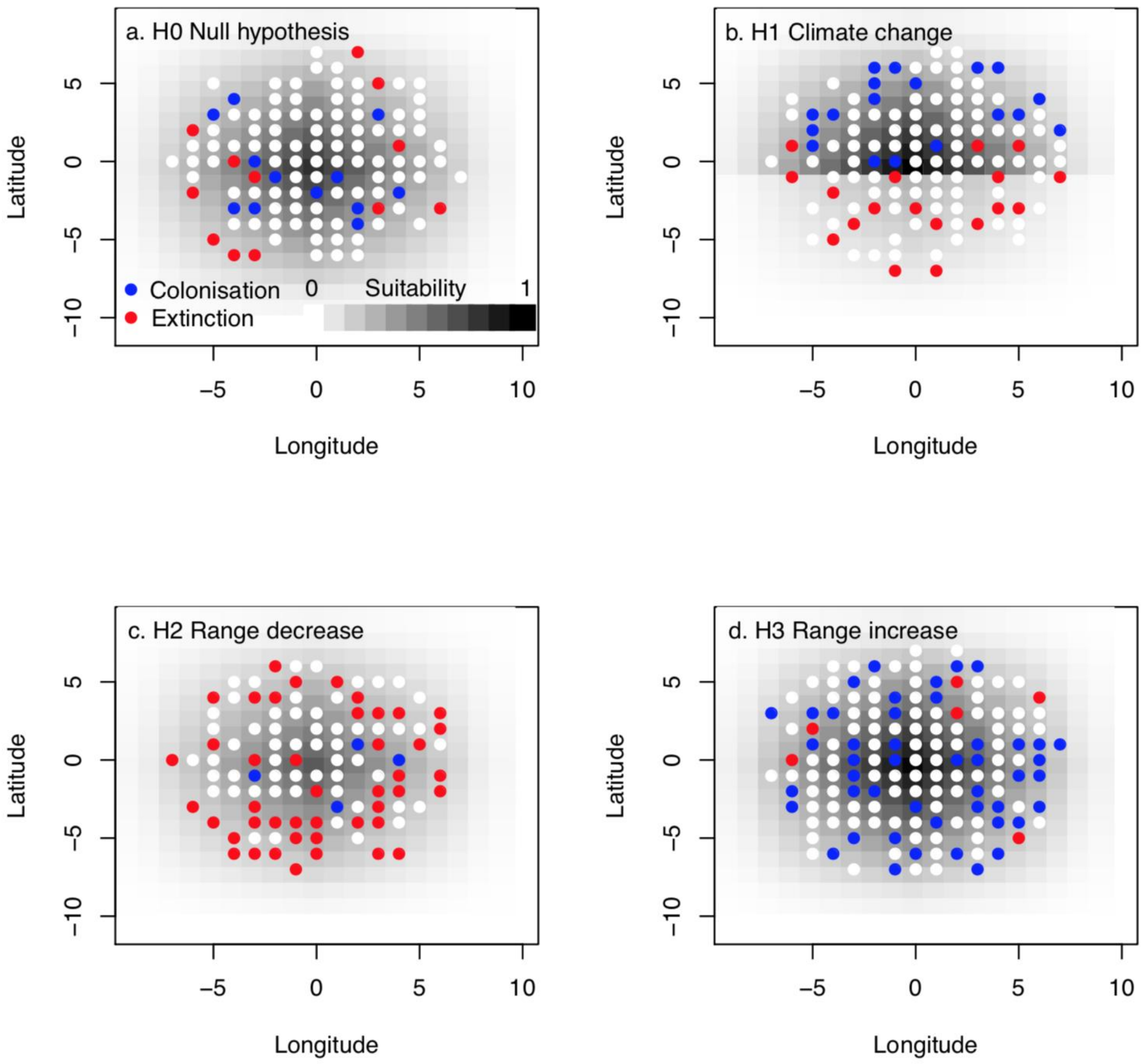

Supplementary Figure S2. Hypothetical situation where colonisations and extinctions are affected by a) random processes, where extinctions are more likely occur at the range edges and colonisations closer to the core of the range due to suitability gradient of the species, b) increased suitability on the cold side of the species' range and decreased suitability on the warm side of species' range due to climate change, c) general reduction or d) increase in suitability for species' occurrence

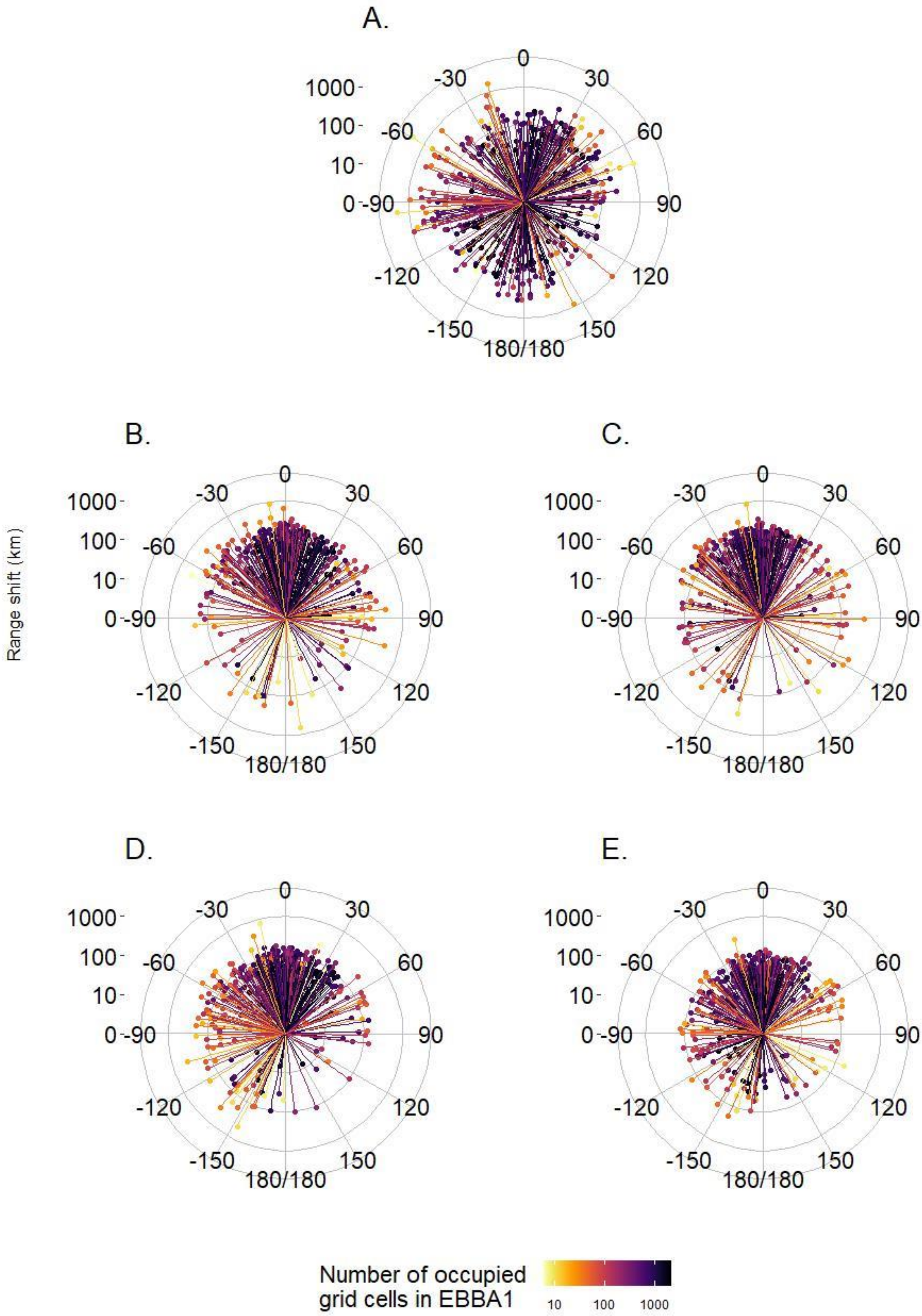

Supplementary Figure S3: Observed (A) and predicted (B – E) shifts in the distance and direction of the ranges of 378 species of European breeding birds between the periods 1985–1988 and 2013–2017 using the four different approaches to fitting SDMs. Observed range shifts (A.) are based on the

species' range data from the European Breeding Bird Atlases (EBBAs). Predicted range shifts are from species distribution models (SDMs) fitted to species data related to the 1985–1988 atlas and projected to the 2013–2017 period. Predicted range shifts derive from SDMs fitted using the best set of climate variables for each individual species (B.), the best set of climate variables from across all species (C.), the best set of climate variables for each individual species and land cover variables (D.), the best set of climate variables from across all species and land cover variables (E.) Each line represents a single species. The centre of each polar plot represents either the observed (A.) or predicted (B.- E.) centre of gravity of the 1985–1988 range for each species. Lines show the distance and direction between the observed or predicted 1985–1988 centre of gravity and the equivalent 2013–2017 range. Lines are coloured by the number of 50 x 50 km UTM grid cells a species occupied in 1985–1988 (i.e., EBBA1). Source data are provided as a Source Data file.

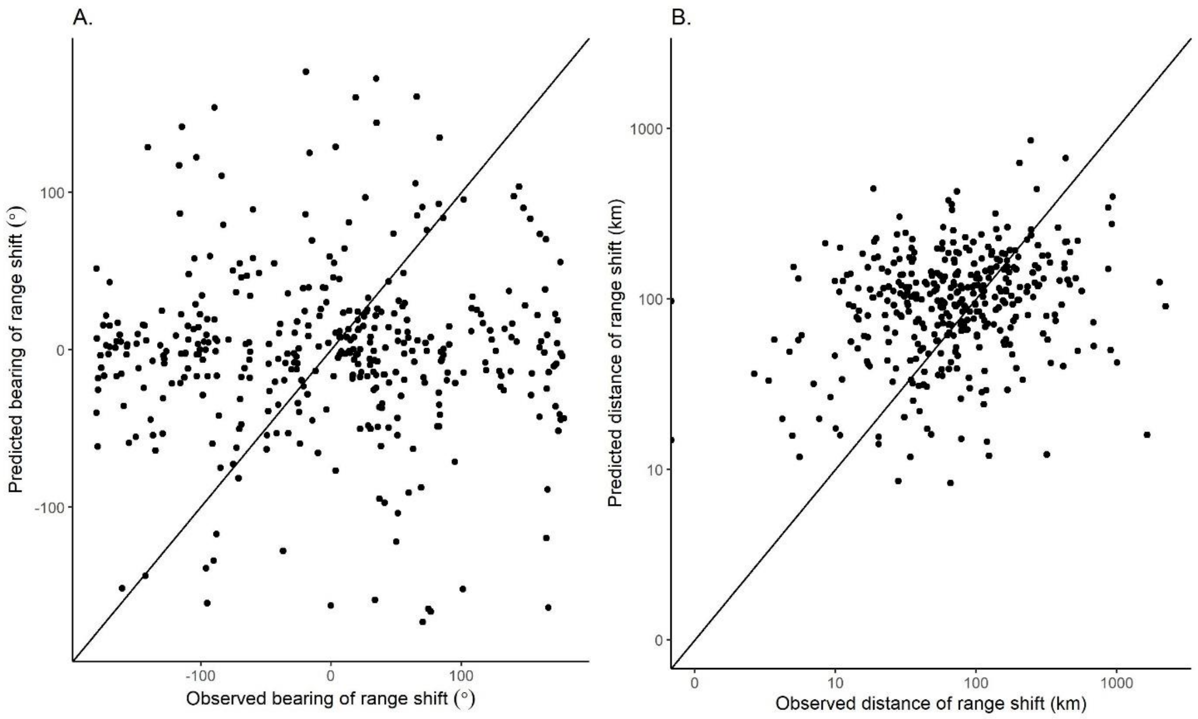

Supplementary Figure S4. Observed versus predicted bearing (A.) and distance (B.) of the range shifts of 378 species of European breeding birds between 1985–1988 and 2013–2017. Range shifts are measured as the direction and distance between the centre of gravity of either the observed or median predicted range using 40 bioclimate SDMs. Predictions are derived from SDMs fitted using the best set of climate predictors for each species. Black lines indicate a one-to-one relationship. Source data are provided as a Source Data file.

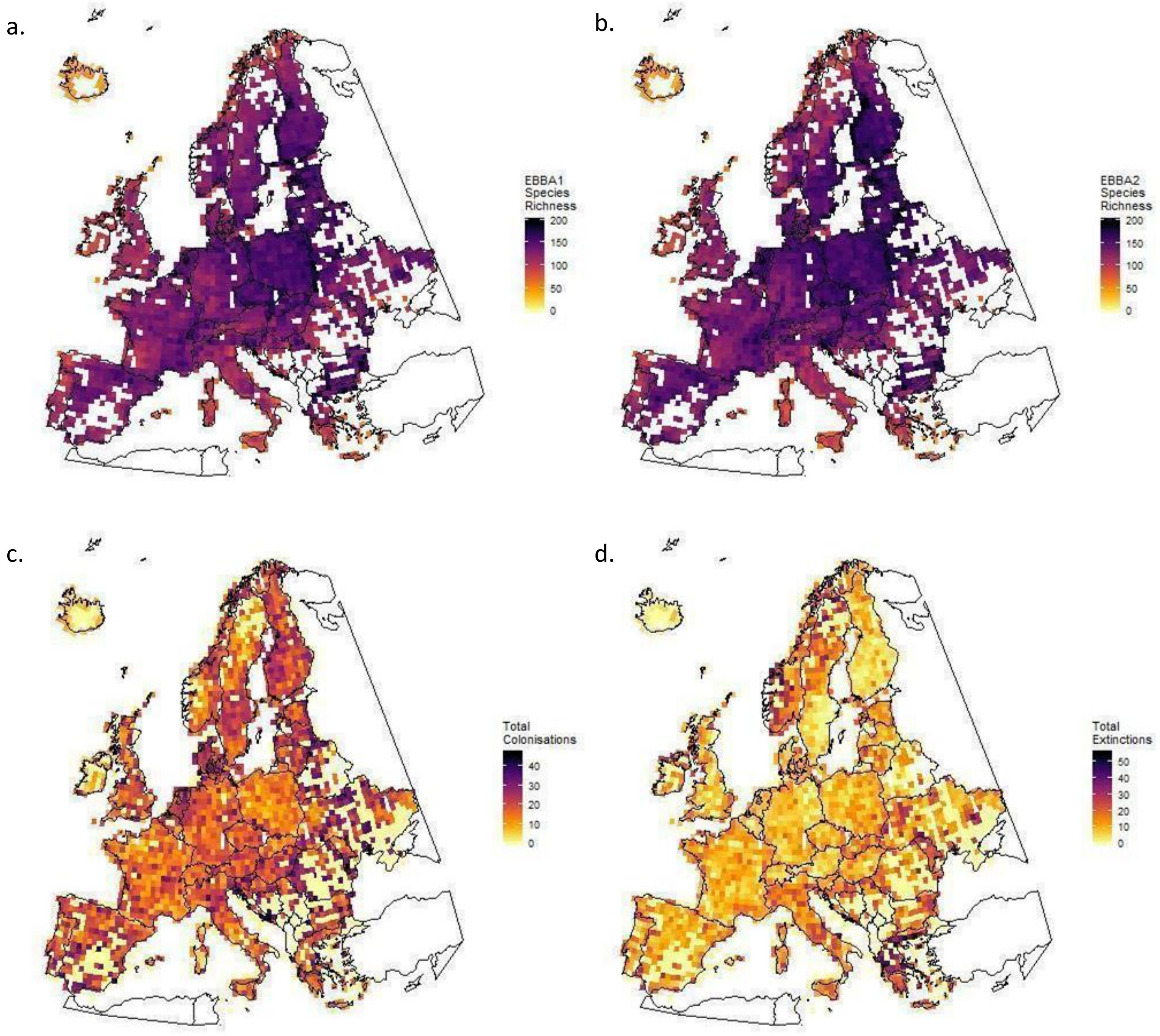

Supplementary Figure S5: Species richness for EBBA 1 (a) and EBBA 2 (b). (c) shows total number of colonisations, whilst (d) shows total number of extinctions across all species observed between the two atlases. Source data are provided as a Source Data file.

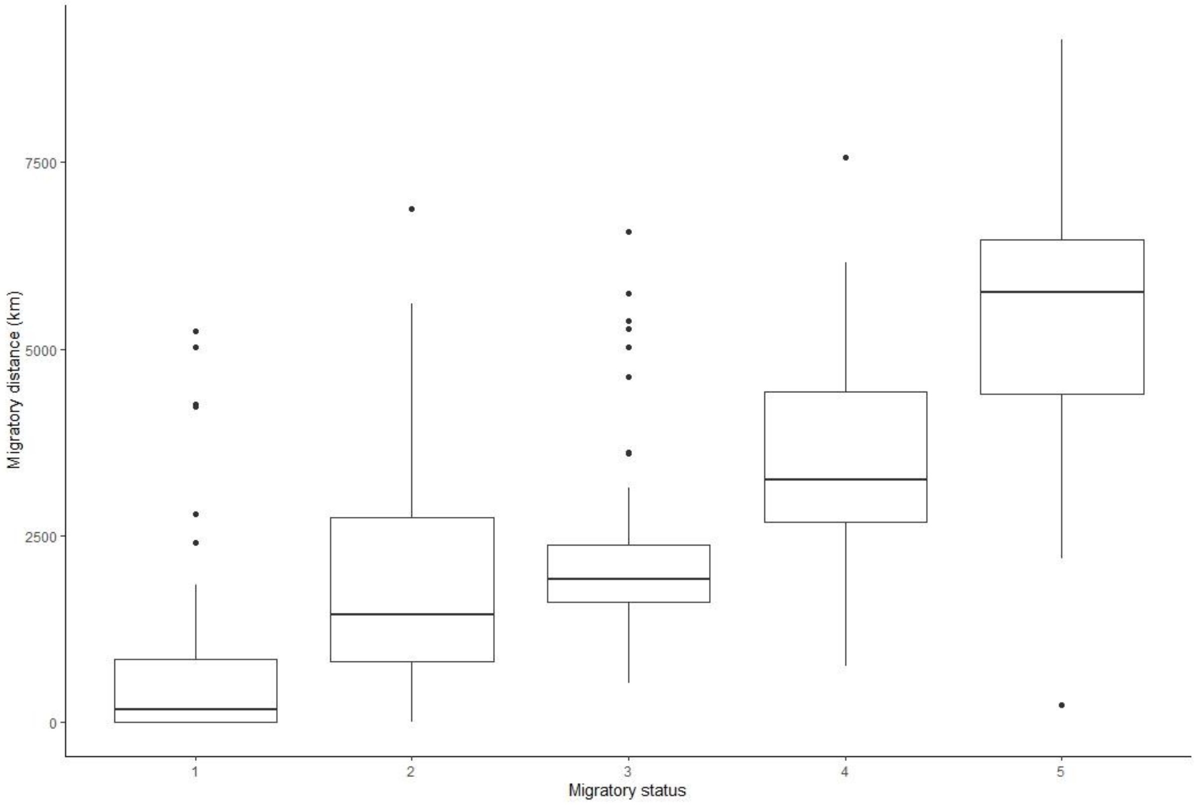

Supplementary Figure S6: Migratory distances for each migratory behaviour category for 378 species of European breeding birds. Migratory distances are measured as the distance between the centre of gravity of species' breeding and non-breeding ranges as defined by BirdLife International [1]. Migratory behaviour categories are defined on a five-point scale ranging from complete resident (1) to obligate long-distance migrant (5). The centre line indicates the median, the box limits indicate the 25th and 75th percentiles, whiskers indicate 1.5 x the interquartile range, and the points indicate outliers. Source data are provided as a Source Data file.

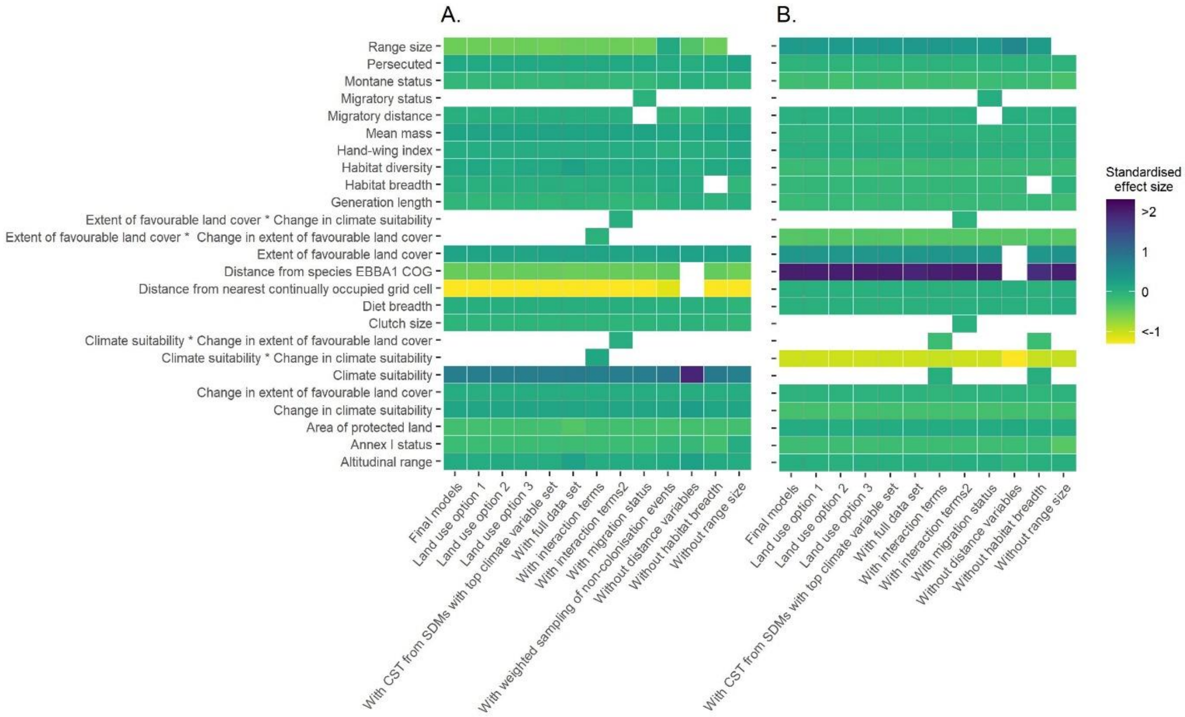

Supplementary Figure S7: Standardised coefficients from sensitivity analyses of MCMC generalised linear mixed models of the colonisation (A) and the local extinction (B) events of European breeding birds between 1985–1988 and 2013–2017. All models accept those fitted without a term for habitat breadth used data on the colonisation or extinction events for 336 species. The model without habitat breadth used data from 378 species. Colours indicate the mean effect size produced by averaging 100 separate MCMCglmms. To standardise coefficient values, all predictors were z-transformed. Columns refer to the 12 variations on the colonisation models and the 11 variations on the extinction models presented in the main analysis. The results from the main analysis are also included for reference. Source data are provided as a Source Data file.

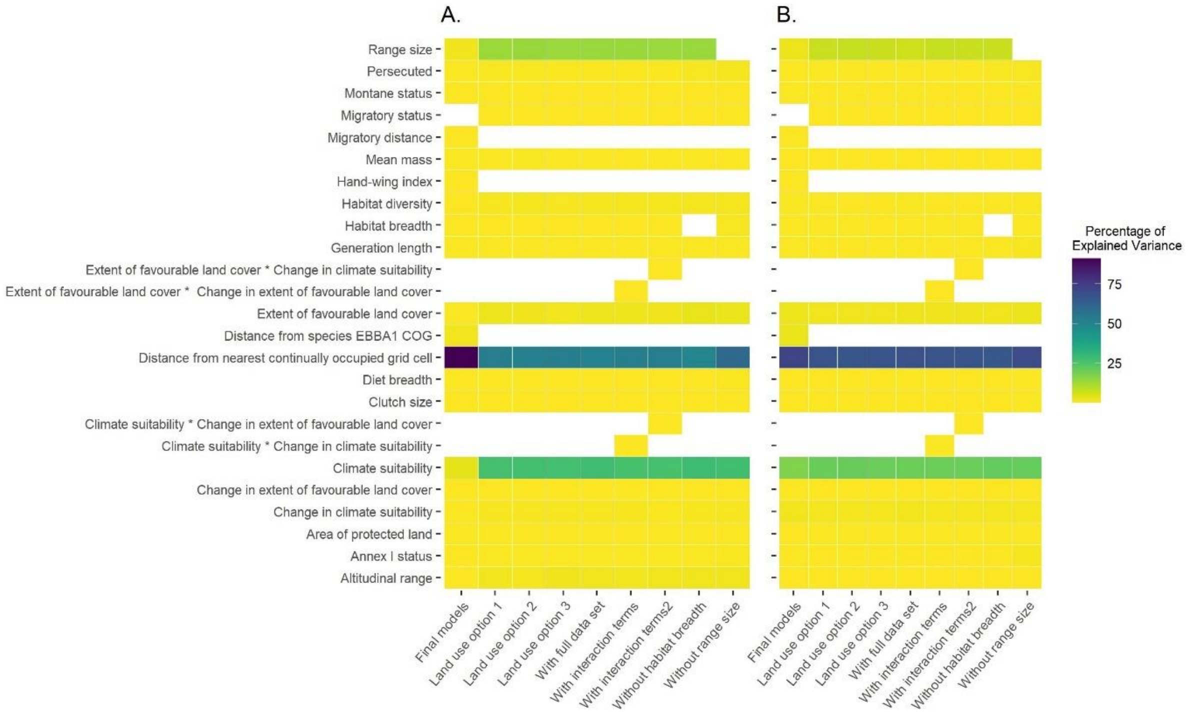

Supplementary Figure S8: Measures of relative variable importance from sensitivity analyses of MCMC generalised linear mixed models of the colonisation (A) and the local extinction (B) events of European breeding birds between 1985–1988 and 2013–2017. All models accept those fitted without a term for habitat breadth used data on the colonisation or extinction events for 336 species. The model without habitat breadth used data from 378 species. Colours indicate the mean percentage of explained variance produced by averaging 100 separate MCMCglmms. Columns refer to the 12 variations on the colonisation models and the 11 variations on the extinction models presented in the main analysis. The results from the main analysis are also included for reference. Source data are provided as a Source Data file.

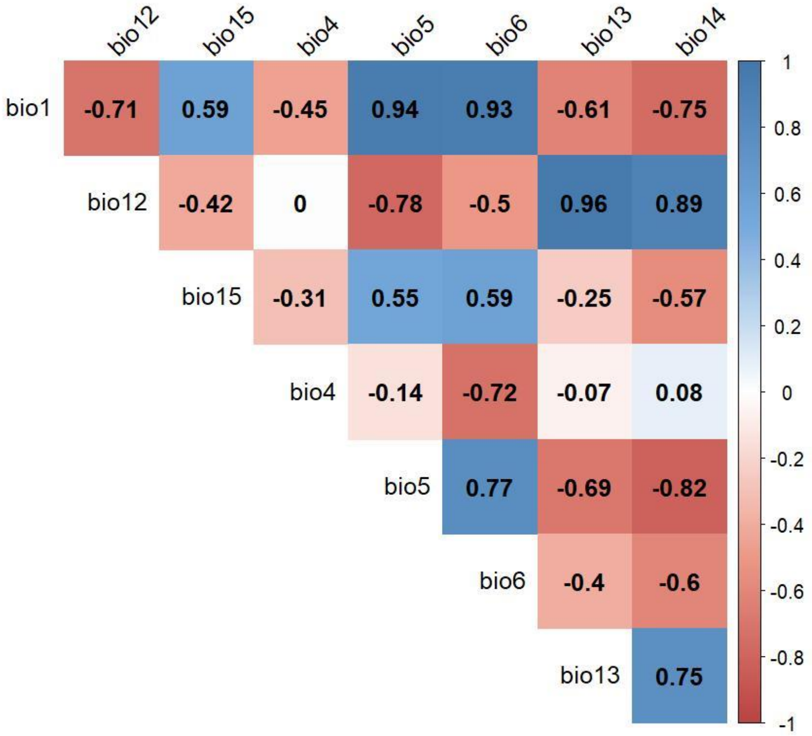

Supplementary Figure S9: Pairwise correlation matrix for the eight climate variables considered in the variable selection process. BIO1 = Annual Mean Temperature, BIO4 = Temperature Seasonality (standard deviation ×100), BIO5 = Max Temperature of Warmest Month, BIO6 = Min Temperature of Coldest Month, BIO12 = Annual Precipitation, BIO13 = Precipitation of Wettest Month, BIO14 = Precipitation of Driest Month, BIO15 = Precipitation Seasonality (Coefficient of Variation). Values indicate Pearsons correlation coefficient. Source data are provided as a Source Data file.

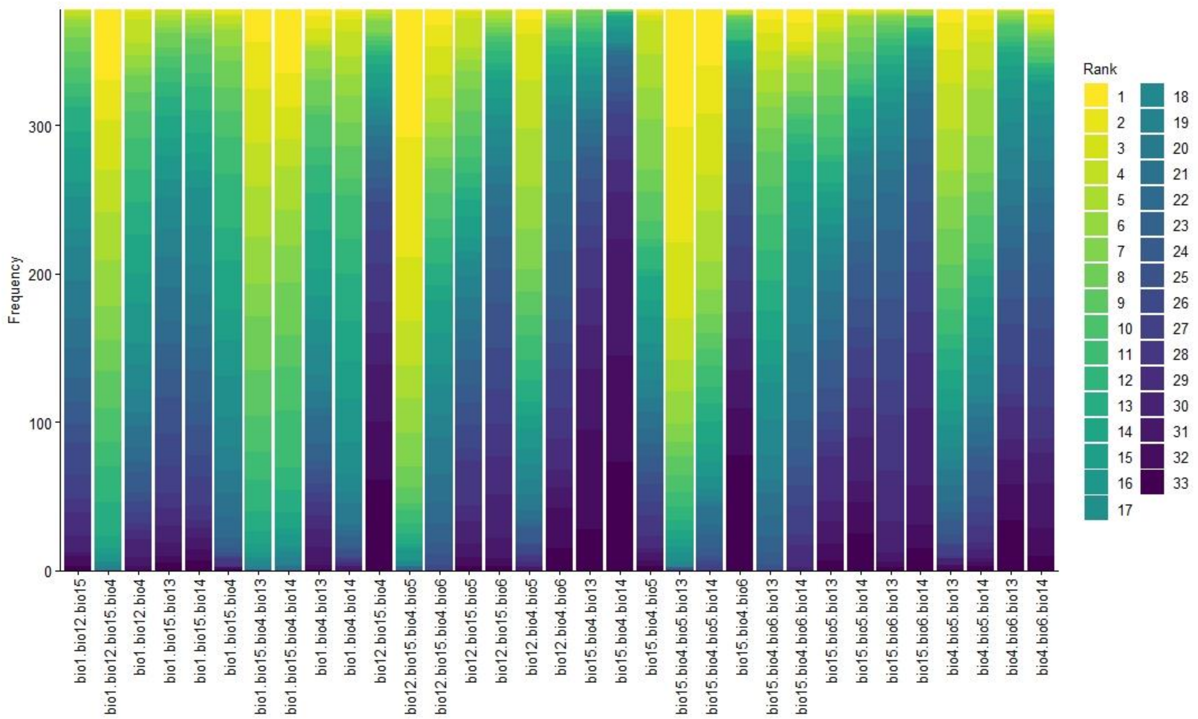

Supplementary Figure S10: SDM performance (measured and ranked using AIC) of 33 different climate variable sets for 378 species of European breeding birds. All three-, four- and five-way combinations of eight climate variables were considered, where variable pairwise correlations did not exceed Pearson R^2 = 0.7. Climate variables include: BIO1 = Annual Mean Temperature, BIO4 = Temperature Seasonality (standard deviation ×100), BIO5 = Max Temperature of Warmest Month, BIO6 = Min Temperature of Coldest Month, BIO12 = Annual Precipitation, BIO13 = Precipitation of Wettest Month, BIO14 = Precipitation of Driest Month, BIO15 = Precipitation Seasonality (Coefficient of Variation). Source data are provided as a Source Data file.

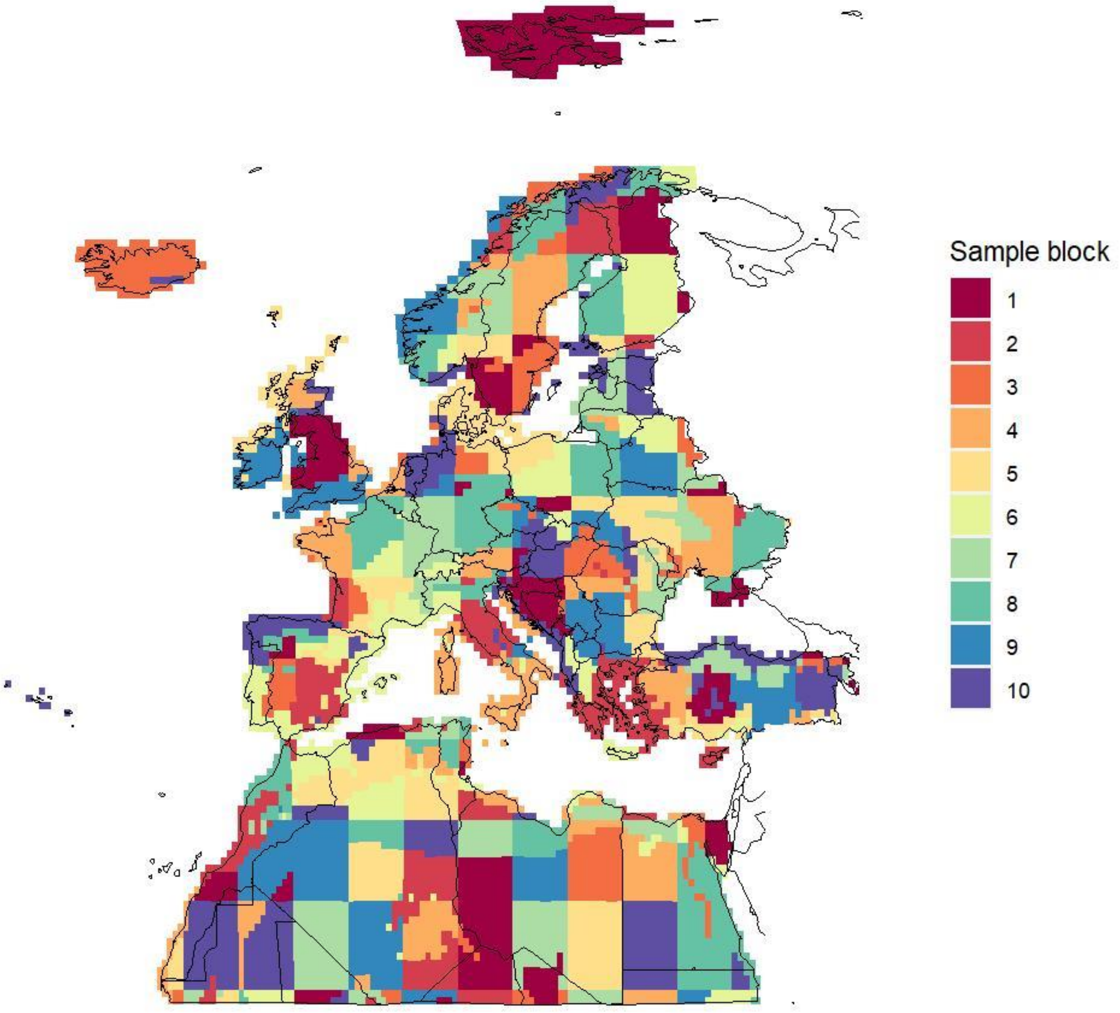

Supplementary Figure S11: Sample blocking used to account for SAC within the SDMs. Here the combined area of Europe, Turkey, and north Africa has been divided into 10 sampling blocks according to the ecoregion to which they belong and their bioclimate. This sampling procedure is designed so that mean bioclimate is similar across all blocks but that the full range of bioclimates is covered within each block. Source data are provided as a Source Data file.

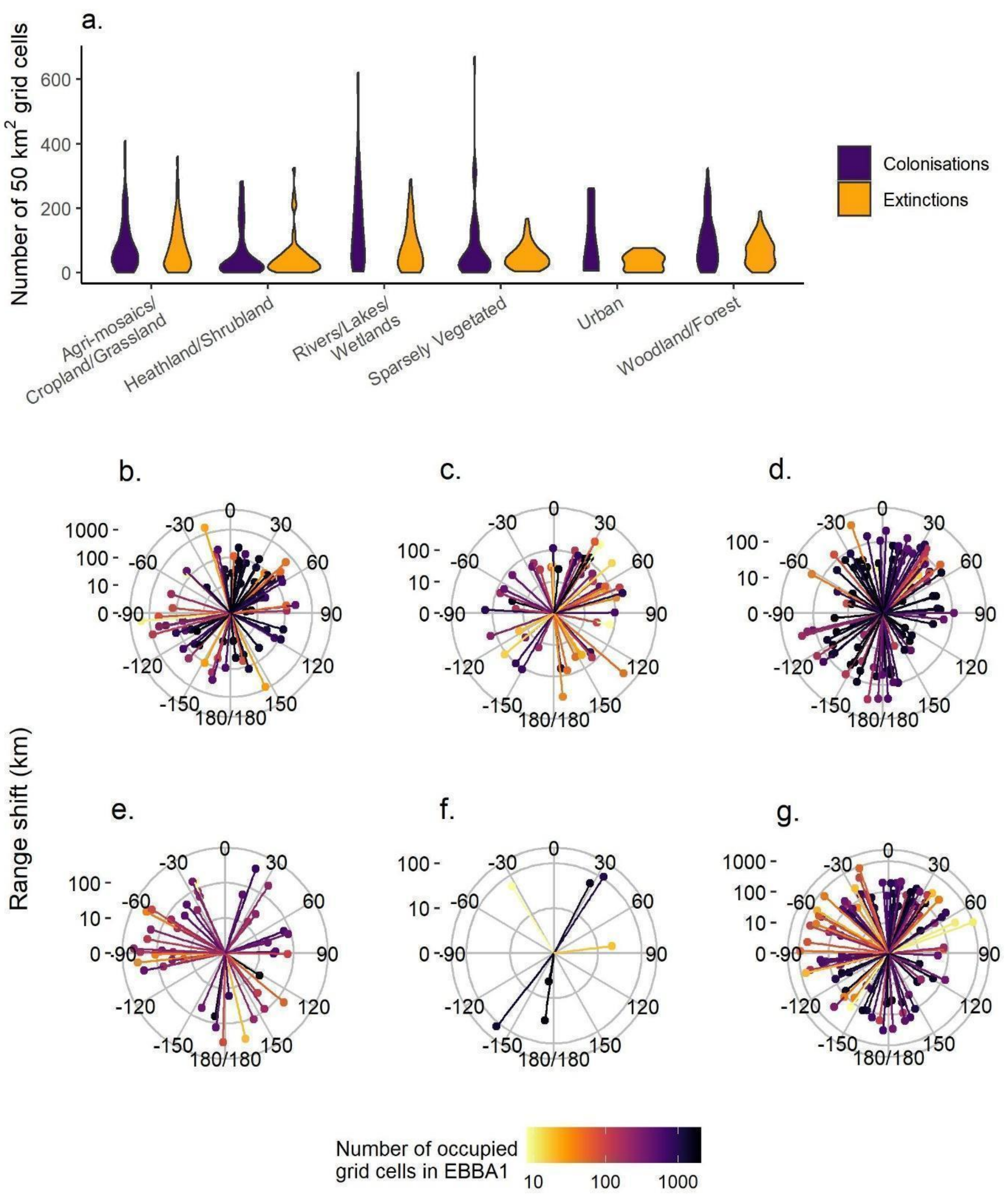

Supplementary Figure S12: Species' range shifts by habitat association. A) kernel probability density plot of the numbers of colonisation and extinction events between 1985–1988 and 2013–2017 for 378 species of European breeding birds associated with different MAES habitat classifications. The observed shifts in the distance and direction of species' ranges are shown for species associated with b) agri-mosaics/cropland/grassland c) heathland/shrubland, d) rivers/lakes/wetlands, e) sparsely vegetated, f) urban, and g) woodland/forest habitats. Each line represents a single species. The centre of each polar plot represents the observed centre of gravity of the 1985–1988 range for each species. Lines show the distance and direction to the observed centre of gravity of the 2013–2017 range. Lines are coloured by the number of cells a species is recorded as present in the 1985–1988 period. Source data are provided as a Source Data file.

**Supplementary Table S1:** Mean and standard deviation (±S.D.) of the performance of species distribution models (SDMs) fitted with different for 378 species of European breeding birds. For each species and each predictor variable set, ten models were fitted for each of the four types of SDMs, producing 40 models overall. Source data are provided as a Source Data file.				
Model	Species-specific climate variable set	Overall climate variable set	Species-specific climate variables with land cover	Overall climate variables with land cover
Overall	0.947 (± 0.040)	0.943 (± 0.046)	0.957 (± 0.033)	0.950 (± 0.360)
GLM	0.947 (± 0.043)	0.944 (±0.045)	0.951 (±0.041)	0.923 (± 0.065)
GAM	0.945 (±0.050)	0.938 (± 0.066)	0.949 (± 0.054)	0.949 (± 0.050)
GBM	0.948 (±0.037)	0.948 (±0.036)	0.960 (±0.031)	0.959 (±0.032)
RF	0.949 (±0.046)	0.951 (±0.039)	0.966 (±0.029)	0.967 (±0.027)

References

1. BirdLife International and NatureServe. Bird species distribution maps of the world. Version 6.0. *Cambridge, UK: BirdLife International* (2016).
