# Supplementary material for: Local colonisations and extinctions of European birds are poorly explained by changes in climate suitability
Source: Nat Commun. 2023 Jul 20;14:4304. doi: 10.1038/s41467-023-39093-1 (PMC10359363; doi:10.1038/s41467-023-39093-1)
Supplement: Supplementary file 3 — Description of Additional Supplementary Information [file 41467_2023_39093_MOESM3_ESM.docx]

**Local colonisations and extinctions of European birds are poorly explained by changes in climate suitability**

**Description of Additional Supplementary Files**

**File Name: Supplementary Information**

Description: Supplementary Figures S1 – S12 and Supplementary Table S1

**File Name: Source Data**

Description: Source data for Figures 1,2, and 3, Supplementary Figures S1, S3- S12, and Supplementary Table S1

Figure 1: Observed and predicted range shifts of 378 species of European breeding birds between EBBA 1 (1985 - 1988) and EBBA 2 (2013 - 2017).

Figure 2: Standardised coefficients and percentage of variance explained from MCMC generalised linear mixed models of the colonisation and extinction events of 336 species of European breeding birds between 1985 - 1988 and 2013 - 2017.

Figure 3: Observed changes in species’ ranges between 1985 - 1988 and 2013 - 2017 and predicted changes in climate suitability for the same time period for four species of European breeding birds.

Supplementary Figure S1: Mean Temperature over the study area between 1960 and 2015

Supplementary Figure S3: Observed and predicted shifts in the distance and direction of the ranges of 378 species of European breeding birds between the periods 1985–1988 and 2013–2017 using the four different approaches to fitting SDMs.

Supplementary Figure S4: Observed and predicted bearing and distance of the range shifts of 378 species of European breeding birds between 1985 - 1988 and 2013-2017.

Supplementary Figure S5: Species richness, and colonisation and extinction patterns across Europe

Supplementary Figure S6: Migratory distances for each migratory behaviour category for 378 species of European breeding birds. Supplementary Figure S7: Standardised coefficients from MCMC generalised linear mixed models of the colonisation and extinction events of European breeding birds between 1985-1988 and 2013-2017.

Supplementary Figure S8: Percentage of variance explained from MCMC generalised linear mixed models of the colonisation and extinction events of European breeding birds between 1985-1988 and 2013-2017.

Supplementary Figure S9: Pairwise correlation matrix for the eight climate variables considered in the variable selection process.

Supplementary Figure S10: SDM performance (measured using AIC) of 33 different climate variable sets for 378 species of European breeding birds.

Supplementary Figure S11: Example of sample blocking for species distribution modelling
Supplementary Figure S12: Observed species' range shifts by habitat association.

Supplementary Table S1: Mean and standard deviation (±S.D.) of the performance of species distribution models (SDMs) measured using AUC fitted with different for 378 species of European breeding birds.

**File Name: Supplementary Data**

Supplementary Data 1

Description: Species’ trait data. References for data sources can be found in the separate sheet. Note traits were collated for 378 species of European breeding bird, but habitat breadth data were only available for 336 species. Sources of species data can be found in the References tab.
