# Supplementary material for: Local colonisations and extinctions of European birds are poorly explained by changes in climate suitability
Source: Nat Commun. 2023 Jul 20;14:4304. doi: 10.1038/s41467-023-39093-1 (PMC10359363; doi:10.1038/s41467-023-39093-1)
Supplement: Supplementary file 5 — Reporting Summary [file 41467_2023_39093_MOESM5_ESM.pdf]

nature portfolio

Corresponding author(s): Christine Howard

Last updated by author(s): Apr 26, 2023

Reporting Summary

Nature Portfolio wishes to improve the reproducibility of the work that we publish. This form provides structure for consistency and transparency in reporting. For further information on Nature Portfolio policies, see our Editorial Policies and the Editorial Policy Checklist.

Statistics

For all statistical analyses, confirm that the following items are present in the figure legend, table legend, main text, or Methods section.

n/a	Confirmed	
☐	☒	The exact sample size (n) for each experimental group/condition, given as a discrete number and unit of measurement
☐	☒	A statement on whether measurements were taken from distinct samples or whether the same sample was measured repeatedly
☐	☒	The statistical test(s) used AND whether they are one- or two-sided<br>*Only common tests should be described solely by name; describe more complex techniques in the Methods section.*
☐	☒	A description of all covariates tested
☐	☒	A description of any assumptions or corrections, such as tests of normality and adjustment for multiple comparisons
☐	☒	A full description of the statistical parameters including central tendency (e.g. means) or other basic estimates (e.g. regression coefficient) AND variation (e.g. standard deviation) or associated estimates of uncertainty (e.g. confidence intervals)
☐	☒	For null hypothesis testing, the test statistic (e.g. F, t, r) with confidence intervals, effect sizes, degrees of freedom and P value noted<br>*Give P values as exact values whenever suitable.*
☐	☒	For Bayesian analysis, information on the choice of priors and Markov chain Monte Carlo settings
☒	☐	For hierarchical and complex designs, identification of the appropriate level for tests and full reporting of outcomes
☐	☒	Estimates of effect sizes (e.g. Cohen's d, Pearson's r), indicating how they were calculated

Our web collection on statistics for biologists contains articles on many of the points above.

Software and code

Policy information about availability of computer code

Data collection	Microsoft Excel 2016 was used for the collation of a species trait data table.
Data analysis	All data analysis was performed in R Version 3.6.1 and require the 'MCMCglmm (2.33)', 'phytools (0.7-80)', 'parallel (4.0.3)', 'stringr (1.4.0)', 'ape (5.6-2) and 'reshape2 (1.4.4)' R packages. Custom code is available at https://doi.org/10.5281/zenodo.7862772.

For manuscripts utilizing custom algorithms or software that are central to the research but not yet described in published literature, software must be made available to editors and reviewers. We strongly encourage code deposition in a community repository (e.g. GitHub). See the Nature Portfolio guidelines for submitting code & software for further information.

Data

Policy information about availability of data

All manuscripts must include a data availability statement. This statement should provide the following information, where applicable:

- Accession codes, unique identifiers, or web links for publicly available datasets
- A description of any restrictions on data availability
- For clinical datasets or third party data, please ensure that the statement adheres to our policy

The data from the first European Breeding Bird Atlas are available from GBIF (https://doi.org/10.15468/adtfvf). The data from the second European Breeding Bird Atlas area available for free from https://ebba2.info/data-request/. Registration will be required for data download. CRU climate data are available from https://crudata.uea.ac.uk/cru/data/hrg/. ESA CCI land cover data are available from https://www.esa-landcover-cci.org/?q=node/1. ETOPO2 altitude data are available from http://www.ngdc.noaa.gov/mgg/global/etopo2.html. WDPA data are available from https://www.protectedplanet.net/). The species trait data compiled in this

study are provided in the Supplementary Information. Source data are provided as a Source Data file.

Human research participants

Policy information about studies involving human research participants and Sex and Gender in Research.

Reporting on sex and gender	NA
Population characteristics	NA
Recruitment	NA
Ethics oversight	NA

Note that full information on the approval of the study protocol must also be provided in the manuscript.

Field-specific reporting

Please select the one below that is the best fit for your research. If you are not sure, read the appropriate sections before making your selection.

☐ Life sciences ☐ Behavioural & social sciences ☒ Ecological, evolutionary & environmental sciences

For a reference copy of the document with all sections, see nature.com/documents/nr-reporting-summary-flat.pdf

Ecological, evolutionary & environmental sciences study design

All studies must disclose on these points even when the disclosure is negative.

Study description	Here, we use data on changes in the breeding distributions of 378 species of European birds over a 30-year period, to quantify the role of a suite of environmental covariates, including climate and land use, along with functional traits, in driving changes in species' distributions. Using these two atlases we identify the local colonization and extinction events that have occurred across Europe during the past 30 years. We collate data on environmental conditions (including climate, elevation, land cover) and species' traits (including mass, clutch size, protection status) from a range of published sources. We then use species distribution models (SDMs) to assess if observed changes in species ranges can be attributed to recent changes in climate. Finally, we use Bayesian phylogenetic mixed models (MCMCglmms) to identify the relative role of the extrinsic environment and species traits in determining the occurrence of local colonization and extinction events.
Research sample	Data on the distributions of 378 European breeding birds were obtained from two Europe-wide distribution atlases (EBCC). These atlases provide records of each species' occurrence across Europe in circa 2,819 50 x 50 km squares of a modified Universal Transverse Mercator (UTM) grid. Data in the first atlas were collected during the 1980s (mainly 1985–1988), and in the second during the 2010s (mainly 2013–2017).
Sampling strategy	For this study we used all data on the local colonization and extinction events of breeding birds across Europe, available from the European Bird Census Council. After excluding some species, for methodological reasons described below, we used data of the occurrence of 378 species in 2,117 50 x 50 km grid cells.
Data collection	Species distribution data and environmental trait data were downloaded as .csv or .txt files, which were read directly into R for subsequent exploration and analysis. Species' trait data were collated from multiple sources, and compiled into a single table using Microsoft Excel 2016.
Timing and spatial scale	Data on the local colonization and extinction events of European breeding birds were obtained from the EBCC in March 2020. These data are derived from two consecutive breeding bird atlases, with data for the first atlas collected during the 1980s (mainly 1985–1988), and in the second during the 2010s (mainly 2013–2017). These atlases provide records of each species' occurrence across Europe for the two periods.
Data exclusions	Data on local colonizations and extinctions are available for 625 species, however, we only included 378 species in the first part of our analysis. These species were excluded as they were considered either invasive or marine, and therefore changes in their distributions can not be attributed to the processes we were examining. We also excluded species where there was taxonomic uncertainty between the two atlases (i.e. from the lumping or splitting of species). Finally, we excluded any species with a limited distribution (i.e. less than 10 data points) and for which we would be able to fit useful models of their distribution. For the second part of the analysis, we excluded a further 42 species due to incomplete covariate data. All of these exclusions are described in our manuscript.
Reproducibility	For both the SDMs and the MCMCglmms, we repeated the model fitting process with ten different subsets of the data and tested their performance on a withheld semi-independent subset of the data. To ensure wider reproducibility of our analysis, all code, including package versions, are available on GitHub (https://github.com/christinehoward399).
Randomization	The analysis was performed using all available data for the local colonization and extinction events for 378 species of European breeding birds. For the fitting of species distribution models, data were split into ten sampling blocks based on ecoregion data. For

the fitting of MCMCglmms, a random sampling approach was used for the selection of 'absences'.

Blinding: Blinding was not relevant for our study as we only used data from published sources.

Did the study involve field work? ☐ Yes ☒ No

Reporting for specific materials, systems and methods

We require information from authors about some types of materials, experimental systems and methods used in many studies. Here, indicate whether each material, system or method listed is relevant to your study. If you are not sure if a list item applies to your research, read the appropriate section before selecting a response.

Materials & experimental systems

n/a	Involved in the study	
☒	☐	Antibodies
☒	☐	Eukaryotic cell lines
☒	☐	Palaeontology and archaeology
☒	☐	Animals and other organisms
☒	☐	Clinical data
☒	☐	Dual use research of concern

Methods

n/a	Involved in the study	
☒	☐	ChIP-seq
☒	☐	Flow cytometry
☒	☐	MRI-based neuroimaging
